# Factors influencing haze formation and corneal flattening, and the impact of haze on visual acuity after conventional collagen cross-linking: a 12-month retrospective study

**DOI:** 10.1186/s12886-021-02066-3

**Published:** 2021-08-23

**Authors:** Anita Csorba, Kinga Kránitz, Péter Dormán, Andrea Popper-Sachetti, Huba Kiss, Irén Szalai, Zoltán Zsolt Nagy

**Affiliations:** 1 Department of Ophthalmology, Semmelweis University, Budapest, Hungary; 2 Department of Clinical Ophthalmology, Semmelweis University, Budapest, Hungary

**Keywords:** Keratoconus, Conventional cross-linking, Densitometry, Corneal haze, Visual outcome

## Abstract

**Background:**

Our aim was to determine associations of pachymetry, keratometry, and their changes with haze formation and corneal flattening after collagen cross-linking, and to analyse the relationship between postoperative haze and visual outcome.

**Methods:**

Retrospective analysis was performed on 47 eyes of 47 patients with keratoconus using the Pentacam HR Scheimpflug camera before and 1, 3, 6 and 12 months after cross-linking. Corneal backscattered light values in grey scale unit were recorded in the anterior, center and posterior corneal layers and in four concentric rings. Surface area- and thickness-corrected grey scale unit values were assessed with an additional calculation. Friedman test with post hoc Wilcoxon signed-rank test was used to analyse changes in visual acuity, pachymetry, keratometry and densitometry. Spearman’s rank correlation test was used to detect correlations of haze formation and corneal flattening with pachymetry, keratometry and their postoperative change. Generalized estimating equations analysis was used to investigate the influence of densitometry values on postoperative visual acuity after controlling for the effect of preoperative keratometry.

**Results:**

One year after treatment, significant flattening was observed in maximum and mean keratometry readings (*p* < 0.001). Significantly increased densitometry values were observed in three central rings compared to baseline (post hoc *p* < 0.0125). According to receiver operating characteristic curve, densitometry value of the anterior layer of 0–2 mm ring was the most characteristic parameter of densitometry changes after cross-linking (area under the curve = 0.936). Changes in haze significantly correlated with preoperative maximum keratometry (*R* = 0.303, *p* = 0.038) and with the changes in maximum keratometry (*R* = -0.412, *p* = 0.004). Changes in maximum keratometry correlated with preoperative maximum keratometry (*R* = -0.302, *p* = 0.038). Postoperative haze had a significant impact on uncorrected and best corrected distance visual acuity (β coefficient = 0.006, *p* = 0.041 and β coefficient = 0.003, *p* = 0.039, respectively).

**Conclusions:**

Our findings indicate that in more advanced keratoconus more significant corneal flattening effect parallel with haze formation can be observed after cross-linking. Despite significant reduction of keratometry, postoperative corneal haze may limit final visual acuity.

## Background

Keratoconus is a bilateral, asymmetric corneal disorder, which leads to progressive protrusion and thinning of the cornea including irregular astigmatism and deterioration of visual acuity [1]. The only effective treatment which can prevent the progression is the minimal-invasive corneal collagen cross-linking (CXL) therapy. Combination of UV-A light and photosensitising riboflavin produces reactive oxigen species, which induces crosslinks between collagen fibrils. Photopolymerization of collagen monomers increases corneal rigidity after treatment [2]. Results of the first clinical study performing CXL treatment on human corneas were published by Wollensak et al. in 2003 [3]. Since then, several long-term clinical studies [4–9] have shown promising clinical outcomes in terms of safety and efficacy.

Corneal haze induces increased backward corneal light scattering, which can be measured by Pentacam HR [10]. Since ’Cornea Densito’ software is available, the degree and location of corneal opacities can be quantified objectively in concentric rings and in different depth layers of the cornea. Several studies have demonstrated increased densitometry values after CXL in keratoconus patients [11–14], mainly in the anterior and middle layers and central rings. Previous studies have already evaluated correlations between densitometry values and postoperative outcomes [15, 16], however no preoperative predictive factors of postoperative corneal haze formation have been yet identified after conventional CXL.

The purpose of the current study was to characterize changes in visual acuity and of corneal haze, curvature, and thickness during a one-year follow-up period after CXL treatment in keratoconus patients. Besides, we aimed to identify the relationship of pachymetry, keratometry, and their postoperative changes with corneal flattening and haze formation, and analyse the associations between postoperative haze and visual outcome.

## Methods

### Patients and study design

This retrospective study was conducted at the Department of Ophthalmology, Semmelweis University with the approval of the Semmelweis University Regional and Institutional Committee of Sciences and Research Ethics. Each participant was informed about the treatments, and written consent was obtained from all patients. All examinations were carried out in accordance with the tenets of the Declaration of Helsinki.

Fourty-seven patients with progressive keratoconus underwent conventional CXL between 2017 and 2018 were enrolled. The surgical intervention was indicated when ectatic progression i.e. increase of maximum keratometry values (≥ 1.0 diopter in 1 year) and/or loss of vision (loss of ≥ two lines of best corrected distance visual acuity in 1 year) was decetected [17]. Patients with age under 18 years, preoperative corneal scarring, previous history of corneal hydrops, prior keratitis, history of any eye injury or corneal thickness below 400 μm were excluded. All pre-, and postoperative data were collected and analysed retrospectively.

### Surgical procedure

Conventional corneal CXL was performed by the same surgeon (Z.Zs.N.) according to Dresden protocol [3]. Oxybuprocaine eye drops (Benoxi®, Unimed Pharma, Bratislava, Slovakia) were instilled preoperatively. Following mechanical removal of epithelial layer in a central diameter of 8 mm, 0.1 % riboflavin droplets (Medio-Haus Medizinprodukte GmbH, Rostock, Germany) were administered topically in every 2 min for 30 min. The cornea was then exposed to UV-A light from a 1-cm distance with a wavelength of 370 ± 5 nm (CSO Vega CMB X Linker, CSO Scandicci, Firenze, Italy) at an irradiance of 3 mW/cm^2^ intensity for the next 30 min with continued instillation of riboflavin in every 2 min. Finally, topical antibiotic drops (5 mg/ml levofloxacin) were instilled and a bandage was placed on. In the early postoperative period, all patients were examined the day after the surgery, when the bandage was removed, and 1 week later after it, when complete corneal re-epithelization was confirmed. Antibiotic drops (5 mg/ml levofloxacin) were instilled five times daily during the first week postoperatively. After complete re-epithelizaiton, in the following one month, topical corticosteroid (1 mg/ml fluorometholone) was administered 4 times per day.

### Examinations

Ophthalmological examinations included uncorrected (UCDVA) and best corrected distance visual acuity (BCDVA) assessment (measured with Snellen charts and converted to logMAR values) with recording spherical equivalent (SEQ) followed by slit-lamp biomicroscopy and Scheimpflug imaging performed preoperatively and at 1, 3, 6 and 12 months after CXL procedure. Pentacam HR Scheimpflug rotating camera (Pentacam HR, Oculus Optikgeräte GmbH, Wetzlar, Germany) was used to measure the thinnest corneal thickness (ThCT), the maximum keratometry values (K_max_), mean keratometry values (K_mean_) and the corneal densitometry values. Only measurements with good image quality – „OK” was shown in quality specification window – were accepted.

Cornea Densito module shows a chart with the average densitometry values of the cornea in grey scale unit (GSU; 0–100 light scattering; 0: maximal trasparency/optically clear cornea; 100: minimal transparency/total corneal opacification) in four concentric rings (0–2 mm; 2–6 mm; 6–10 mm; 10–12 mm) and in three different layers. The „Anterior” layer represents the front 120 μm, while the „Posterior” refers the last 60 μm of corneal thickness from epithelial to endothelial layer. The „Center” layer gives the values between these two layers, thus, the thickness of this layer is variable in every patients. „Total” layer represents the average optical density of the full corneal thickness. Additionally, since different rings have different surface areas and thicknesses, raw densitometry data in GSU were converted to GSU/cubic millimeters (GSU/mm^3^), as previously published by Nemeth et. al [13]. First, we received values in GSU/mm^2^ with dividing raw GSU data of each concentric ring by its area (3.141 mm^2^ for ring 0–2 mm; 25.132 mm^2^ for ring 2–6 mm; 50.265 mm^2^ for ring 6–10 mm; 34.557 mm^2^ for ring 10–12 mm and 78.539 mm^2^ for total diameter). Then values of different rings in GSU/mm^2^ were divided by the thickness of the appropriate layer in mm (0.12 mm for the anterior layer and 0.06 for the posterior layer). The thickness of the center layer was calculated by substracting 120 and 60 μm from total thickness which was recorded from „Pachy apex” window (the X and Y coordinates of this point are 0.00) and then it was converted to mm. With this additional calculation we got surface area- and thickness-corrected densitometry values (cGSU) in GSU/mm^3^.

### Statistical analysis

The statistical analysis was performed using IBM® SPSS® Statistics for Windows, version 25.0 (IBM Corp., Armonk, N.Y., USA). Kolmogorov-Smirnov test was used to assess the normality of the variables. Based on the results of normality test, the changes in UCDVA, BCDVA, SEQ, keratometry, pachymetry and densitometry were evaluated using nonparametric Friedman test. Where Friedman test resulted in statistical significance, post hoc pairwise comparison was implemented using Wilcoxon signed-rank test for comparison of each time point to the baseline (i.e. baseline vs. 1 month, baseline vs. 3 months, baseline vs. 6 months and baseline vs. 12 months). As these four analyses were conducted for each repeated testing procedure, Bonferroni-adjusted significance level was applied for each comparison resulting in a significance level at *p* < 0.0125. Receiver operating characteristic (ROC) curve was plotted to determine the most characteristic densitometry data of corneal changes at 12-month visit. Comparison of ROC curves was performed to test the statistical significance of the difference between the areas under the curves (AUC) with the method of DeLong et al. using MedCalc®, version 19.4.1. (MedCalc Software Ltd., Ostend, Belgium) [18]. Spearman’s rank correlation test was used to find relationships between pachymetry, keratometry, and their postoperative changes and in haze formation and corneal flattening. The associations of postoperative visual acuity (dependent variable of interest) with the densitometry values (independent variable) 1 year after surgery was assessed with generalized estimating equations (GEE). Preoperative maximum keratometry was incorporated as a covariate in the regression model to adjust for potentional cofounding. The level of significance was considered at *p* < 0.05 in all analysis, except the post hoc Wilcoxon test.

## Results

Fourty-seven eyes of 47 patients including 35 male and 12 female subjects with keratoconus underwent conventional CXL therapy were involved in this study. The mean age was 26.72 ± 6.03 (range, 18–38) years before CXL. All involved subjects were Caucasian. The cohort comprised 21 eyes (44.7 %) in stage I, 19 eyes (40.4 %) in stage II and 7 eyes (14.9 %) in stage III according to Amsler-Krumeich classification of keratoconus severity [19]. No ophthalmological complications developed due to the CXL surgery during the follow-up period.

Friedman test revealed statistically significant changes in SEQ postoperatively (*p* < 0.001). Post hoc evaluation showed significant improvement 6 and 12 months after CXL (*p* < 0.001 and *p* < 0.002, respectively). According to Friedman test, statistically significant changes were found both in UCDVA and in BCDVA after CXL (both *p* < 0.001). Regarding UCDVA, significant difference was found at 1 month compared to baseline (post hoc *p* = 0.005). At 1-, 3-, and 6-month visit, BCDVA was lower than preoperatively (post hoc *p* > 0.0125), but the difference was statistically significant only also at the first postoperative month, as revealed by post hoc Wilcoxon signed-rank test (post hoc *p* = 0.005). At 12 months, improvement in was not significant compared to the baseline value neither in UCDVA, nor in BCDVA (post hoc *p* > 0.0125).

There were significant changes both in K_max_ and K_mean_ values and in ThCT values postoperatively, as determined by Friedman test (both *p* values < 0.001). Post hoc analysis revealed that at 1 month there was a significant increase in K_max_ value from preoperative 56.41 ± 5.41 D to 57.15 ± 5.74 D (post hoc *p* < 0.001), after which keratometry began to decrease. One year after CXL, K_max_ readings decreased significantly to 55.35 ± 5.18 D compared to baseline (post hoc *p* < 0.001). Change in K_mean_ showed similar tendency with significant changes at 3, 6 and 12 months (post hoc *p* < 0.001). Average decrease was -1.05 ± 1.13 D in K_max_ value (ΔK_max_) and it was -0.71 ± 0.78 D in K_mean_ during follow-up period. Compared to the preoperative value, ThCT decreased significantly in all postoperative month (all post hoc *p* values < 0.001). One year after treatment, ThCT decreased by -15.5 ± 15.19 μm. UCDVA, BCDVA, SEQ, pachymetry and keratometry values at various times are listed in Table 1.

**Table 1:** Uncorrected- and best corrected distance visual acuity (UCDVA and BCDVA, logMAR), spherical equivalent (SEQ, D), thinnest corneal pachymetry (ThCT, µm), maximum and mean keratometry (K_max_ and K_mean_, D) at different visits

Variables	Preoperative	Postoperative			
		1 months	3 months	6 months	12 months
BCDVA (logMAR)	0.16 ± 0.2	0.23 ± 0.21^†^	0.18 ± 0.19	0.18 ± 0.18	0.13 ± 0.2
UCDVA (logMAR)	0.56 ± 0.38	0.64 ± 0.35^†^	0.56 ± 0.35	0.55 ± 0.34	0.51 ± 0.32
SEQ (D)	-3.77 ± 3.74	-2.97 ± 2.61	-2.95 ± 2.65	-2.65 ± 2.65^†^	-2.59 ± 2.58^†^
ThCT (µm)	476.49 ± 34.57	444.3 ± 38.88^†^	451.39 ± 37.75^†^	455.11 ± 36.52^†^	460.94 ± 35.74^†^
K_max_ (D)	56.41 ± 5.41	57.15 ± 5.74^†^	56.01 ± 6.03	55.75 ± 5.67	55.35 ± 5.18^†^
K_mean_ (D)	47.47 ± 3.49	47.54 ± 3.87	46.93 ± 3.64^†^	46.81 ± 3.61^†^	46.76 ± 3.31^†^

Data are shown as mean ± standard deviation

^†^Statistically significant difference compared to baseline (Bonferroni-adjusted post hoc *p*-value < 0.0125)

Table 2 shows the mean cGSU values at different follow up visits. Statistically significant changes were found in densitometry values of anterior, central and total layer of 0–2 mm, 2–6 mm and 6–10 mm ring after CXL according to Friedman test (all *p* values < 0.001). Upon further testing using the Wilcoxon test, regarding the concentric rings of the cornea, in 0–2 mm and in 2–6 mm rings, cGSU values were significantly increased at all postoperative visits in anterior, center and total layers (all post hoc *p* values < 0.0125). In 6–10 mm ring, significantly increased cGSU was found in the anterior layer at 1 and 3 months (both post hoc *p* < 0.001) and in center and total layers at all follow-up visits (post hoc *p* < 0.0125). There was a peak in densitometry values 3 months after CXL in the most central zone and after 1 month in the 2–6 mm and 6–10 mm rings. After the peak, densitometry values decreased. Considering the different corneal layers, Friedman test showed significant changes in total densitometry of anterior, center and total layers (all *p* < 0.001). Post hoc analysis revealed that densitometry values of these layers were significantly increased at all postoperative visits (all post hoc *p* < 0.001) except the total densitometry of center layer at 12 months (post hoc *p* = 0.079). Densitometry values in the remaining zones did not change significantly after CXL (all post hoc *p* > 0.0125) (Table 2).

**Table 2:** Corrected densitometry values in grey scale unit per cubic millimeter (GSU/mm^3^) at all postoperative months

Variables		Preoperative	Postoperative				p*	p**
			1 months	3 months	6 months	12 months		
Anterior	0–2 mm	79.29 ± 6.75	107.45 ± 17.86†	113.55 ± 21.8†	104.27 ± 16.86†	100.64 ± 14.06†	< 0.001	< 0.001
	2–6 mm	8.45 ± 0.66	11.07 ± 1.35†	10.71 ± 1.12†	9.76 ± 1.13†	9.4 ± 0.84†	< 0.001	< 0.001
	6–10 mm	3.39 ± 0.37	3.83 ± 0.63†	3.56 ± 0.41†	3.42 ± 0.37	3.42 ± 0.36	< 0.001	= 0.17
	10–12 mm	6.54 ± 1.87	6.23 ± 2.05	6.27 ± 2.09	6.27 ± 1.74	6.45 ± 2.23	= 0.895	
	Total	2.63 ± 0.24	3.17 ± 0.37†	3.13 ± 0.36†	2.93 ± 0.33†	2.87 ± 0.31†	< 0.001	< 0.001
Center	0–2 mm	18.74 ± 2.41	25.49 ± 5.93†	26.17 ± 8.23†	22.98 ± 5.23†	22.26 ± 9.68†	< 0.001	< 0.001
	2–6 mm	1.98 ± 0.27	2.63 ± 0.53†	2.49 ± 0.58†	2.24 ± 0.36†	2.12 ± 0.33†	< 0.001	< 0.001
	6–10 mm	0.89 ± 0.13	1.07 ± 0.27†	1.0 ± 0.18†	0.96 ± 1.16†	0.93 ± 0.15†	< 0.001	= 0.003
	10–12 mm	1.83 ± 0.53	1.86 ± 0.46	1.92 ± 0.64	1.94 ± 0.56	1.85 ± 0.5	= 0.119	
	Total	0.66 ± 0.08	0.82 ± 0.18†	0.79 ± 0.17†	0.74 ± 0.13†	0.66 ± 0.08	< 0.001	= 0.079
Posterior	0–2 mm	67.88 ± 9.33	68.98 ± 9.39	71.65 ± 13.7	66.73 ± 9.49	67.68 ± 9.03	= 0.418	
	2–6 mm	8.50 ± 0.78	10.09 ± 9.82	8.61 ± 0.74	8.24 ± 0.82	8.27 ± 0.64	= 0.032	= 0.039
	6–10 mm	4.24 ± 0.63	4.32 ± 0.78	4.23 ± 0.59	4.12 ± 0.51	4.16 ± 0.49	= 0.439	
	10–12 mm	8.43 ± 0.94	8.37 ± 1.99	8.2 ± 1.89	8.43 ± 1.83	8.61 ± 1.96	= 0.451	
	Total	2.86 ± 0.33	2.89 ± 0.38	92.87 ± 0.32	2.8 ± 0.29	2.83 ± 0.26	= 0.659	
Total	0–2 mm	13.25 ± 1.34	17.34 ± 3.92†	18.3 ± 3.92†	16.54 ± 2.81†	15.94 ± 3.02†	< 0.001	< 0.001
	2–6 mm	1.46 ± 0.14	1.89 ± 0.28†	1.88 ± 0.28†	1.65 ± 0.21†	1.58 ± 0.16†	< 0.001	< 0.001
	6–10 mm	0.64 ± 0.07	0.74 ± 0.15†	0.69 ± 0.08†	0.67 ± 0.08†	0.66 ± 0.07†	< 0.001	= 0.007
	10–12 mm	1.27 ± 0.33	1.33 ± 0.42	1.29 ± 0.38	1.29 ± 0.33	1.32 ± 0.38	= 0.473	
	Total	0.47 ± 0.05	0.57 ± 0.1†	0.56 ± 0.08†	0.52 ± 0.07†	0.51 ± 0.06†	< 0.001	< 0.001

Data are shown as mean ± standard deviation

^†^Statistically significant difference compared to baseline (Bonferroni-adjusted *p-*value < 0.0125)

*p-values of Friedman tests

**Post hoc *p*-value of 12-month data compared to baseline

**Fig. 1:**
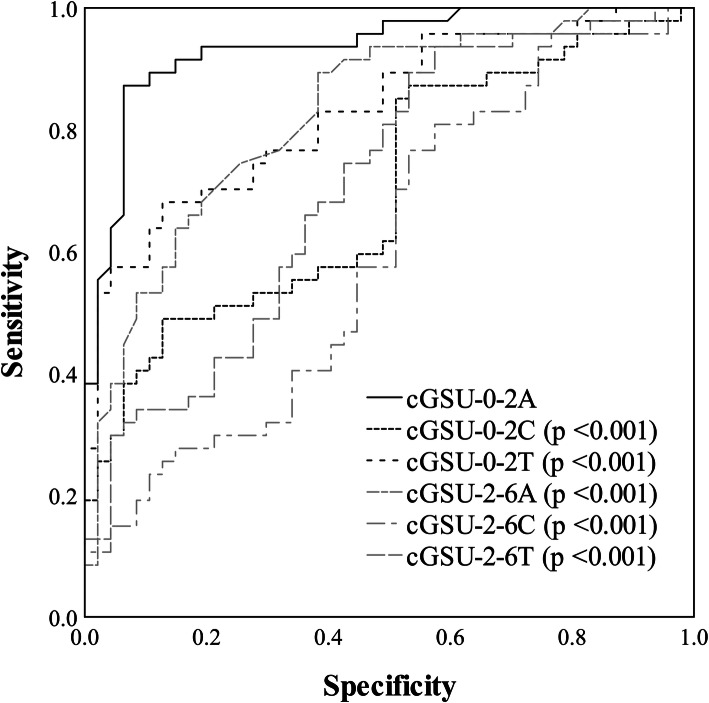
Receiver operator characteristic (ROC) curve analysis of densitometry data in different regions. ROC analysis determined corrected densitometry data in anterior layer of 0–2 mm ring (cGSU-0-2A) as the most sensitive parameter of corneal densitometry changes after cross-linking (CXL). All significantly increased corrected densitometry data at 12 months from the central rings mostly affected by CXL were plotted, i.e. anterior, center and total layers of 0–2 mm and of 2–6 mm rings (cGSU-0-2A, cGSU-0-2C, cGSU-0-2T and cGSU-2-6A, cGSU-2-6C, cGSU-2-6T). Comparison of AUCs showed AUC of cGSU-0-2A was statistically significant compared to other AUCs (*p-*values are shown for corresponding parameters)

Figure 1 shows the results of ROC curve analysis for corrected densitometry values. For ROC analysis, significantly increased corrected densitometry data at 12 months were used from the central rings mostly affected by CXL (anterior, center and total layer of 0–2 mm and of 2–6 mm rings). According to ROC curve, at postoperative 12 months, the corrected densitometry in the anterior layer of the 0–2 mm ring (cGSU-0-2A) was the most characteristic parameter of corneal densitometry changes after CXL (AUC = 0.936). AUC of cGSU-0-2A was statistically significant compared to other AUCs (cGSU-0-2C, *p* < 0.001; cGSU-0-2T, *p* < 0.001; cGSU-2-6A, *p* < 0.001; cGSU-2-6C, *p* < 0.001 and cGSU-2-6T, *p* < 0.001, respectively). Therefore, the data of cGSU-0-2A region were hereinafter used to examine the relationship between preoperative data and densitometry values. Average increase in densitometry of this region was 21.35 ± 14.69 GSU/mm^3^ as compared to the baseline (Fig. 1).

**Fig. 2:**
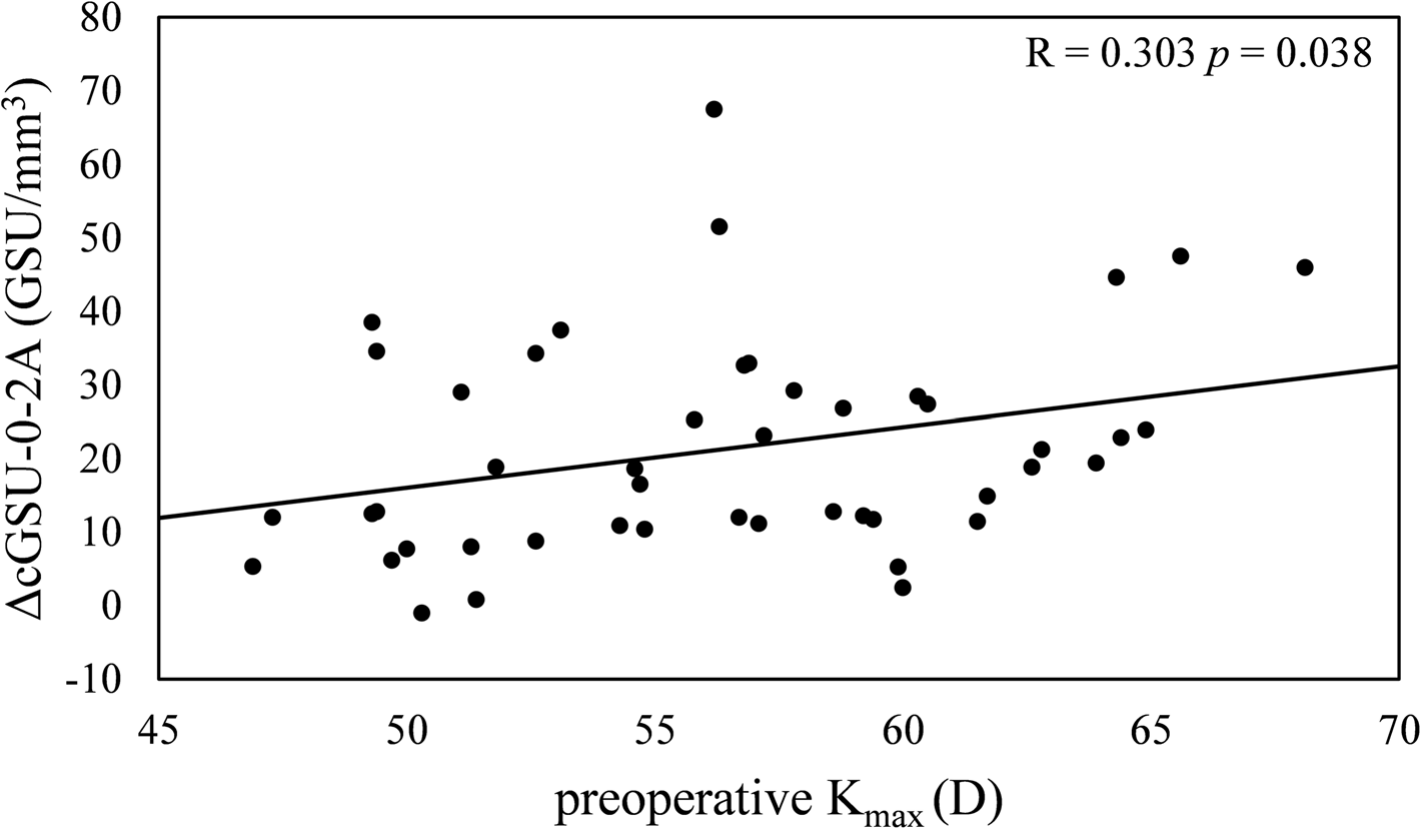
Relationship between the changes in haze (ΔcGSU-0-2A, GSU/mm^3^) and preoperative maximum keratometry (K_max_, D). Statistically significant correlation was found between ΔcGSU-0-2A and preoperative K_max_

**Fig. 3:**
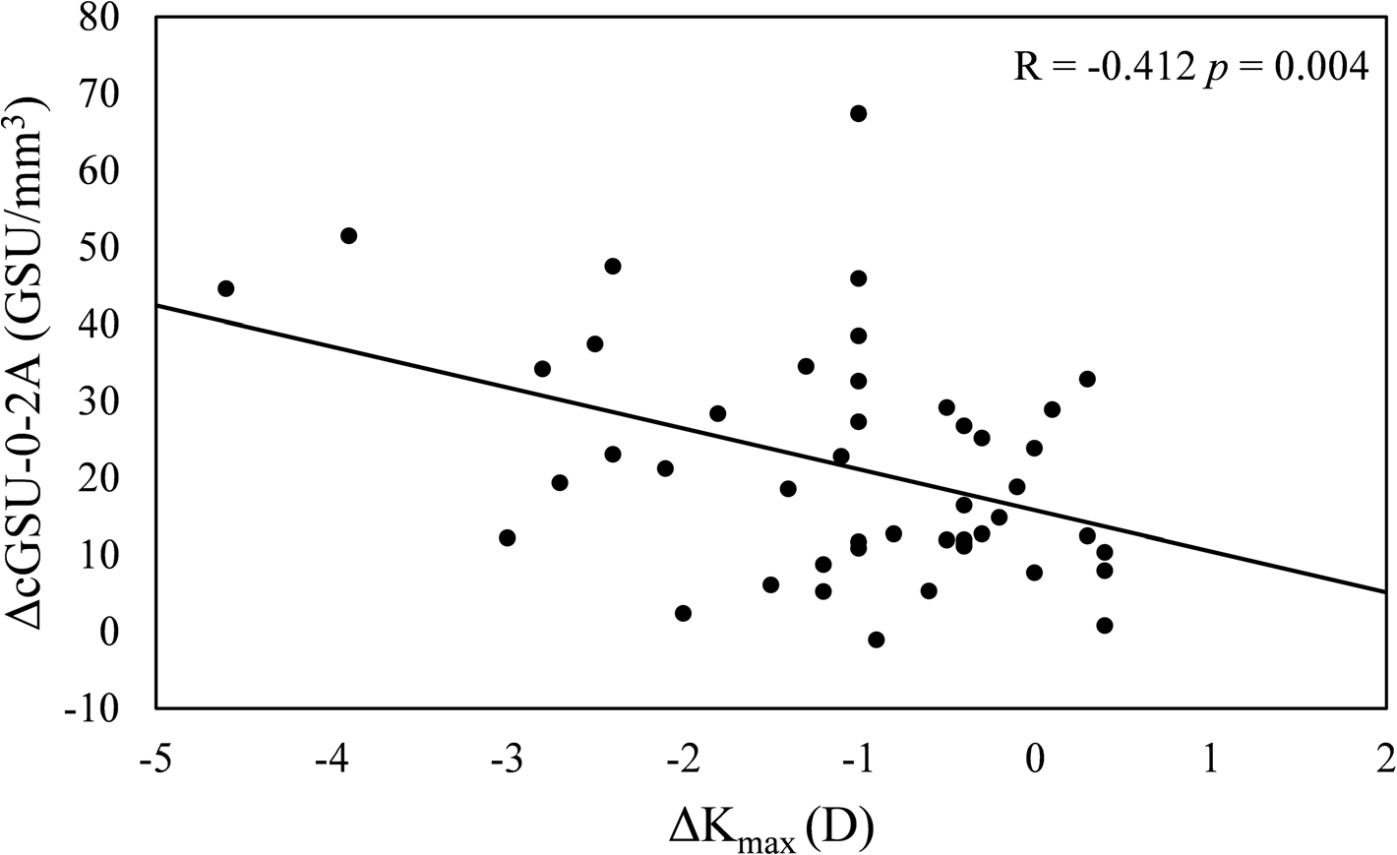
Relationship between changes in densitometry at 12 months (ΔcGSU-0-2A, GSU/mm^3^) and changes in maximum keratometry (ΔK_max_, D). Higher decrease in K_max_ (i.e. corneal flattening) is associated with greater increase of densitometry

**Fig. 4:**
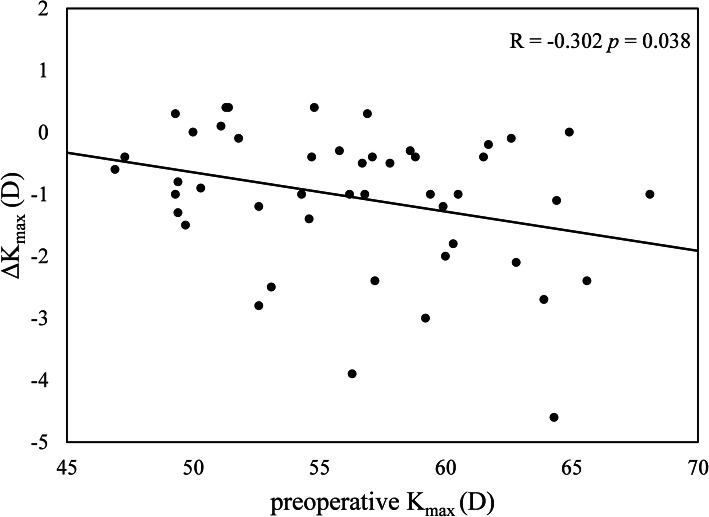
Changes in maximum keratometry (ΔK_max_, D) significantly correlated with preoperative maximum keratometry (K_max_, D)

Considering the relationship between the changes in densitometry (ΔcGSU-0-2A) and the preoperative parameters, one year after CXL, the changes in haze in this region was statistically correlated with preoperative K_max_ (*R* = 0.303, *p* = 0.038) (Fig. 2). At postoperative 12 months, ΔcGSU-0-2A was moderately correlated with ΔK_max_ (*R* = -0.412, *p* = 0.004) (Fig. 3). No correlation was observed between ΔcGSU-0-2A and preoperative ThCT (*R* = -0.022, *p* = 0.885) and changes in thinnest pachymetry (ΔThCT) (*R* = -0.27, *p* = 0.066). Age did not have any effect neither on cGSU-0-2A at postoperative 12 months (*R* = 0.177, *p* = 0.233) nor on ΔcGSU-0-2A (*R* = 0.097, *p* = 0.514). The analysis of the influence of preoperative values on corneal flattening showed significant negative correlation between ΔK_max_ and preoperative K_max_ readings (*R* = -0.302, *p* = 0.038) however ΔK_max_ did not correlated with preoperative ThCT (*R* = 0.094, *p* = 0.53) (Fig. 4).

GEE analysis showed that postoperative densitometry (cGSU-0-2A) has a significant effect both on postoperative UCDVA (β coefficient = 0.006, *p* = 0.041) and postoperative BCDVA (β coefficient = 0.003, *p* = 0.039) after adjusted for the effect preoperative K_max_ readings (β coefficient = 0.026, *p* < 0.001 and β coefficient = 0.018, *p* < 0.001, respectively) (Table 3).

**Table 3:** Results of generalized estimating equations adjusted for preoperative K_max_ readings to determine relationship between postoperative corneal haze (cGSU-0-2 A, GSU/mm^3^) and postoperative uncorrected and best corrected distance visual acuity (UCDVA and BCDVA, logMAR)

	UCDVA		BCDVA	
	β coefficient	*p*-value	β coefficient	*p*-value
cGSU-0-2A	0.006	0.041	0.003	0.039
K_max_	0.026	< 0.001	0.018	< 0.001

## Discussion

Herein in the present study, classical corneal densitometry alterations measured by Cornea Densito software of Pentacam HR after conventional CXL therapy in keratoconus patients during 1-year follow-up were reported. Beside this, the impact of keratometric and pachymetric values on haze formation and corneal flattening were also assessed.

The highly organized structure of corneal tissue is an important factor of corneal transparency [20]. However, corneal collagen cross-linking is considered as a safe technique to stabilize the cornea in progressive keratoconus, it may induce haze formation. Previous confocal microscopic studies [21, 22] found various cellular changes after CXL, which might modify corneal transparency and associated with densitometry changes. In the early postoperative months, keratocyte loss due to apoptosis and edematous, hyper-reflective extracellular stroma can be observed. These alterations may last for 6 months, but generally disappears after 12 months postoperatively, which is followed by resolution of the haze [22].

In our study, we found initial worsening of UCDVA and BCDVA at the first postoperative month, which was followed by an improvement; however, the difference was not statistically significant compared to the preoperative values at 12 months. Changes in K_mean_ and K_max_ readings showed similar trend with steepening at 1 month, after which they began to stabilize and showed significant flattening one year after treatment. Densitometry values were peaked between the first and the third postoperative months, after they began to decrease. Presumably, our findigs are consistent with the aforementioned cellular changes, i.e. CXL-induced stromal modifications cause higher keratometry and densitometry values and worsening of visual acuity in early postoperative months, but later with remodelling process these parameters are stabilized.

In this detailed analysis of corneal densitometry, increased densitometry values were found after CXL in the three central rings (0–2 mm, 2–6 mm, 6–10 mm). Densitometry changes were restricted to the anterior and center layers, while the posterior layer was not affected in any region. These findings supported our expectations, since cross-linking has its predominant effect within 300 μm depth in corneal stroma [23, 24]. According to some previous clinical trials [25, 26] corneal haze rarely lasts longer than 12 months; however, in these studies haze assessment was performed by slit-lamp examination. Grading of postoperative haze was first described by Fantes et al. based on slit-lamp observation of the cornea and rating haziness on a scale from 0 to 4 [27]. Using slit-lamp evaluation, it is generally difficult to detect very subtle changes in haze levels, thus this scoring system for the purpose of statistical analysis is not refined enough. Moreover, grading on this scale is a subjective method with an inherent lack of intra- and interobserver repeatability and reproducibilty [28]. Scheimpflug-camera with add-on densitmetry software gives the possibility of a more sensitive and reproducible method of corneal haze detection. Our results showed that one year after CXL, densitometry values remained elevated in the anterior, center and total layers of 0–2 mm and of 2–6 mm rings, and in the center and total layer of 6–10 mm ring compared to the baseline. This pattern is very similar to those studies in which densitometry changes were detected by Scheimpflug imaging [12–15], and suggest that cellular modifications in the central cornea persist at least one year after treatment.

In the literature there are contradictory data on which corneal region is mostly affected by CXL. Most of the studies describe that the highest densitometry change can be measured in the anterior layer [12, 13], while the main involvement of the center layer has also been reported [15]. It might be because the thickness of center corneal layer is not defined in exact µm in Cornea Densito software of Pentacam HR, and differs in every individual. Therefore, in thicker corneas, the results of center layer represent values measured in a thicker center layer, which may affect the final analysis. Moreover, examined rings have different surface areas, which may influence the obtained results [13]. In our study, we used a new additional calculation for densitometry data decribed by Nemeth et al. [13], with which we got surface area- and thickness-corrected densitometry values. Using surface area- and thickness corrected densitometry values allows values to be evaluated independently of the individual corneal thickness or different surface areas of examined rings. With analysing densitometry using corrected data, our study showed that densitometry alteration in anterior layer of 0–2 mm ring proved to be the most relevant parameter of corneal densitometry changes.

Most of the previous studies investigated the correlations between CXL-induced haze and postoperative outcome [12, 15, 16], while the predictive impact of preoperative parameteres on postoperative degree of haze after conventional CXL has not been defined so far. Pircher et al. [12] reported significantly higher densitometry values in eyes with greater decrease in keratometry readings, but the exact correlation has not been revealed. In our study, authors analysed the influence of relevant preoperative parameters defining the stage of keratoconus, i.e. ThCT and K_max_ on haze formation and corneal flattening. Preoperative K_max_ was the only preoperative factor which correlated significantly with changes in haze and with changes in keratometry. Recently, several studies [29–31] have highlighted the prognostic significance of preoperative keratometry in determining the amount of topographic flattening after CXL. Our study has also demonstrated that a greater flattening might be expected in corneas with higher preoperative keratomtery readings. Ultrastructural findings of keratoconic corneas include high portion of loosely packed and randomly oriented collagen fibrils [32]. Extent of structural alterations varies as the disease progresses [33]. In case of advanced keratoconus, cross-linking effect may penetrate relatively deeper, thus, larger proportion of stroma becomes crosslinked, which may result in greater amount of corneal haze. This could be a reason for more pronounced haze formation after CXL in patients with high keratometry readings and suggests that increased densitometry values might be predictive of effectiveness of CXL in terms of corneal flattening and stiffening.

Patients’ age might have an effect on the densitometry values determined by Pentacam in normal corneas [34]. It also has been described that the corneal clarity depends on the severity of keratoconus: in more advanced keratoconus, due to the increasing corneal damage, higher densitometry values can be measured [35]. Studies examining corneal densitometry changes after CXL in keratoconus differ in involved patients’ ages: some of these reports strictly contain adults over 18 years [12, 15, 36, 37], whereas some of them involved patients under 18 years as well [11, 13, 38], although the presented tendencies in densitometry changes are similar. A recent study showed that in juvenile keratoconus patients significantly higher postoperative haziness can be observed after CXL compared to adults [39]. Our results showed that the postoperative densitometry changes after CXL depend rather on the keratoconus severity, than on the patients’ age, which is in consistent with the available literature [12, 15, 16, 35].

Numerous studies have investigated the visual outcome after conventional cross-linking and reported results are contradictory. Some authors have reported significant improvement of BCDVA [15, 40, 41], while others did not support that [12, 14, 31]. According to some recently available data [42–44], preoperative keratometry has a significant impact on postoperative outcome in terms of visual acuity, i.e. patients with higher preoperative keratometry values are likely to show more improvement in BCDVA. The results of several previous researches suggest that CXL-associated stromal haze does not affect high-contrast visual acuity [12, 14]. In our study, an analysis was performed how UCDVA and BCDVA at 12 months would alter with a one-unit change in densitometry values. Therefore, the impact of densitometry values on UCDVA and BCDVA were assessed via generalized estimating equations. Preoperative keratometry readings were kept in the model to adjust its impact on visual outcome. Using this model, we found that elevated densitometry values negatively affected visual acuity one year after treatment. In our study, improvement was not significant neither in UCDVA nor in BCDVA at the end of follow-up period, although significant corneal flattening was observed. According to our results, it can be concluded that in spite of the fact that CXL treatment decreases keratometry values and may improve vision, loss of transparency may have a limiting effect on improvement of visual acuity.

Currently, there is no generally accepted protocol for the type of postoperatively used topical corticosteroids after CXL. Recent literature suggests that fluorometholone is a commonly used topical corticosteroid after CXL for preventing or minimizing corneal haze formation [13, 16, 25, 45]. However, fluorometholone is a low potent corticosteroid and may increase the risk of corneal haze compared to more potent corticosteroids, e.g. dexamethasone or betamethasone. It has been shown that keratoconic eyes may have predisposition to the development of steroid-induced ocular hypertension using topical dexamethasone after CXL [46]. Although there is no data about changes of intraocular pressure during using less potent steroids after CXL. Theoretically, in more advanced keratoconus, where more significant haze formation may be expected, using of potent topical corticosteroids might be considered, but only with close monitoring of eye pressure. Future studies are required in order to reveal the effects of different topical cortiocosteroids on haze formation after CXL.

The findings of this study reported herein have to be seen in the light of some limitations. First, this was a retrospective study with limited number of patients. Second, the follow-up period was one year, although it can not be ruled out that the degree of corneal haze may change thereafter. These limitations may impact the generalizability of our results to greater population of patients with keratoconus underwent CXL and to those, whom several years have elapsed since the treatment. Some disadvantages of densitometry measurement with Scheimpflug camera must also be mentioned. It has been proven before that the repeatability of densitometry measurement was low in CXL-treated corneas [47], thus, for more exact results, it would have been preferable to use the average value of three measurements at every follow-up time points. Variances in white-to-white distance among patients may lead to the false inclusion of limbus and sclera, thus the presentation of higher densitometry values in peripheral rings [35]. Despite the fact that the instrument provides an objective method for measuring haze, the necessity for such a refined measurement in clinical practice is debatable. Moreover, Cornea Densito software is not available on every Pentacam device since it needs to be installed additionally. Finally, analysing data of separated groups according to the severity of keratoconus migh have influenced our final results. Estimating possible densitometry changes according to different stages based on a detailed classification system (such as ABCD grading system) might be useful for clinicians in predicting postoperative results as well. Future studies may investigate postoperative densitometry changes in exactly defined keratoconus stages.

## Conclusions

In conclusion, with densitometry modul of Pentacam HR, structural corneal changes after conventional cross-linking can be observed even one year after therapy. Our results demonstrated that more pronounced reduction in maximum keratometry values is associated with a greater increase in corneal densitometry and greater flattening effect. Although cross-linking is effective in stabilizing corneal ectasia and reducing keratometry, loss of transparency may limit the improvement of visual acuity.

## Data Availability

The datasets used and/or analysed during the current study are available from the corresponding author on reasonable request.

## Abbreviations

CXL: Cross-linking
UCDVA: Uncorrected distance visual acuity
BCDVA: Best corrected distance visual aquity
SEQ: Sperical equivalent
ThCT: Thinnest corneal thickness
K_max_: Maximum keratometry
K_mean_: Mean keratometry
GSU: Grey scale unit
cGSU: Corrected densitometry values
ROC: Receiever operating characteristic
AUC: Area under curve
GEE: Generalized estimating equations
ΔK_max_: Changes in K_max_ value
cGSU-0-2A: Corrected densitometry in the anterior layer of the 0–2 mm ring
ΔcGSU-0-2A: Changes in corrected densitometry in the anterior layer of the 0–2 mm ring
ΔThCT: Changes in thinnest pachymetry
